# The Effect of Preventing Oxidative Stress and Its Mechanisms in the Extract from *Sonchus brachyotus* DC. Based on the Nrf2-Keap1-ARE Signaling Pathway

**DOI:** 10.3390/antiox12091677

**Published:** 2023-08-27

**Authors:** Meng-Jie Zhang, Wen-Wen Sun, Juan Yang, Dong-Dong Shi, Xiao-Feng Dai, Xiu-Mei Li

**Affiliations:** Key Laboratory of Feed Biotechnology, Ministry of Agriculture and Rural Affairs, Institute of Feed Research of CAAS, Beijing 100081, China

**Keywords:** *Sonchus brachyotus* DC. extracts (SBE), preventing oxidative stress, Nrf2-Keap1-ARE signaling pathway

## Abstract

As the organ with the largest contact area with the outside world, the intestine is home to a large number of microorganisms and carries out the main functions of food digestion, absorption, and metabolism. Therefore, there is a very active metabolism of substances and energy in the gut, which is easily attacked by oxygen free radicals. What is more, oxidative stress can gradually and slowly cause very serious damage to the gut. Hence, maintaining redox balance is essential for maintaining environmental balance in the gut. Our previous studies have demonstrated that the extract of *Sonchus brachyotus* DC. (SBE) has been shown to be capable of repairing oxidative damage, while it has not been demonstrated that it can prevent oxidative stress or how it develops. In this work, we investigated the prevention of oxidative stress and its mechanism in SBE based on the H_2_O_2_-induced oxidative damage model in Caco-2 cells; the results indicate that SBE can reduce the contents of ROS and MDA and increase the activities of SOD and CAT in preventing oxidative stress. Then, at the mRNA and protein level, SBE can up-regulate and down-regulate the expression of related genes (*NFE2L2*, *KEAP1*, *HMOX1*, *NQO1*, *SOD1*, *CAT*, and *GPX1*) and proteins involved in the Nrf2-Keap1-ARE signaling pathway. In conclusion, SBE plays a preventive role in oxidative stress through the Nrf2-Keap1-ARE signaling pathway.

## 1. Introduction

Oxidative Stress (OS) refers to the imbalance between excessive oxidant production and antioxidant defense in the body. It occurs when the net amount of reactive oxygen species exceeds the antioxidant capacity, so oxidative stress is also called ROS–antioxidant imbalance [1]. Reactive oxygen species (ROS) are a series of reactive, oxidizing, oxygen-containing substances, including superoxide anions (O_2_^−·^), hydroxyl radicals (·OH), and hydrogen peroxide, that are produced during the reduction of molecular oxygen in living organisms [2,3]. When unfavorable external conditions produce oxidative stress in the organism and the degree of oxidation exceeds the body’s antioxidant capacity, the concentration of ROS increases, which leads to tissue damage, oxidative damage to DNA, lipid peroxidation, and oxidation of protein [4,5,6]. Mild oxidative stress is regulated by the body’s antioxidant system, which is the body’s first line of defense against oxidative stress, including superoxide dismutase (SOD), glutathione peroxidase (GSH-Px), and catalase (CAT) [7]. A large number of studies have shown that the uncontrolled production and concomitant increase in ROS levels in the body leading to “oxidative stress” is an important contributor to the pathogenic process of many diseases [8].

The gut is the main part of the body for digesting and absorbing nutrients. Among all fully differentiated organs of the human body, intestine cells renew most frequently because they are a cavity organ with the highest circulation space between the body and the outside environment. Intestinal tissue is more vulnerable to oxidative injury than other organs because it forms connections with the external environment and is constantly exposed to foreign substances [9]. Studies have demonstrated that the gastrointestinal tract is a significant generator of reactive oxygen species (ROS) and one of the body’s tissues that is most susceptible to their oxidative attack [10,11]. Reduced immunity and infection with numerous intestinal disorders further affect intestinal oxidative stress. Oxidative stress is linked to a number of gastrointestinal conditions, including gastritis, gastric cancer, IBD, colonic inflammation, and colorectal cancer [12,13,14]. Therefore, it is crucial to lessen the oxidative stimulation of the gut, enhance the function of the intestinal mucosa’s defensive line, and maintain the intestine’s overall health.

Recent studies have shown that plant extracts are emerging as natural, efficient, and safe antioxidants. *Sonchus brachyotus* DC. (*Sonchus brachyotus*), a member of the Sonchus family, is an annual herb that is used to treat severe dysentery, enteritis, and other disorders due to it having the properties of eliminating heat, detoxification, and halting bleeding according to ancient Chinese medicine books [15]. According to previous investigations, the methanol extract of *Sonchus brachyotus* has a considerable ability to scavenge DPPH and ABTS^+^ and exhibit good antioxidant activity in vitro [16]. Meanwhile, in our previous work, we demonstrated that the ethanol extract of *Sonchus brachyotus* can remove the free radicals DPPH and ABTS^+^, and then we revealed the key active ingredients of SBE to be polysaccharides, alkaloids, and polyphenols by analyzing the primary constituents. In addition, the chemical composition of SBE was analyzed by high-performance liquid chromatography [17,18]. In vivo, studies on zebrafish indicated that SBE could repair oxidative damage in the intestine by lowering the levels of oxidative stress indicators (ROS and MDA), raising the activities of antioxidant enzymes, and altering the intestinal flora to resist oxidative stress [18]. However, previous studies have focused on the repair of oxidative stress by SBE, while the mechanism by which SBE protects against oxidative stress remain unclear. Therefore, in this study, we investigated the preventive effect of SBE on the cellular response to oxidative stress based on the H_2_O_2_-induced oxidative stress model in Caco-2 cells.

## 2. Materials and Methods

### 2.1. Chemicals and Reagents

The ROS (Item No.:E004-1-1) assay kit was purchased from Nanjing Jiancheng Bioengineering Institute (Nanjing, China). Malondialdehyde (MDA) (Item No.: S0131S), catalase (CAT) (Item No.: S0051), superoxide dismutase (SOD) (Item No.: S0060), Thiazolyl Blue (MTT), and bicinchoninic acid (BCA) protein assay kits were all purchased from the Beyotime Institute of Biotechnology (Shanghai, China). Cell culture medium DMEM/F12, Fetal Bovine Serum (FBS), penicillin–streptomycin solution, 0.25% trypsin-EDTA, and Phosphate Buffered Saline (PBS) were purchased from Life Technologies Gibco (Beijing, China). Dimethyl sulfoxide (DMSO) and Tween 20 were purchased from Sigma-Aldrich (Shanghai, China). DNase/RNase-free water and PMSF were purchased from Solarbio. TRIzol^®^ reagent, isopropyl alcohol, chloroform, and absolute ethanol were acquired from Sinopharm Chemical Reagent Limited Corporation (Beijing, China). TransStart Green qPCR SuperMix was purchased from TransGen Biotech (Beijing, China). TransScript First-Strand cDNA Synthesis SuperMix was purchased from Abcam (Cambridge, UK). Ammonium persulfate, glycine, and TEMED were purchased from Biosharp. Sodium dodecyl sulfate (SDS) and Tris were purchased from Biotopped (Beijing, China). GAPDH mouse monoclonal antibody (Lot number: SC47724) and HRP-labeled goat anti-mouse IgG (H+L) (Lot number: SC55712) were purchased from SANTA CRUZ BIOTECHNOLOGY (Shanghai, China). Nrf2 mouse monoclonal antibody (Lot number: CAIQ0118101X) and Keap1 mouse polyclonal antibody (Lot number: XAV0219072) were purchased from R&D SYSTEMS (Beijing, China).

### 2.2. Plant Material

The aerial parts of *Sonchus brachyotus* were collected from Binzhou City, Shandong Province, China in 2020. All specimens, which were authenticated by Xiumei Li (Feed Research Institute, Chinese Academy of Agricultural Sciences, Beijing, China), were dried in the shade until the weight remained constant.

### 2.3. Extract Preparation

All dried samples were ground and then passed through a 60-mesh sieve and then ultrasonically extracted according to the conditions of 75% (*v*/*v*) ethanol/water solution, 1:30 (g/mL) ratio of material to liquid, and 700 W ultrasonic power for 30 min. Subsequently, after centrifugation at 5000× *g* for 10 min, the eluate was evaporated by rotary evaporation at 40 ± 2 °C to make a powder out of the liquid plant extract using vacuum freeze-drying technology, which was then stored at 4 °C [19].

### 2.4. Cell Culture

Caco-2 cells were purchased from Beijing Zhongke Quality Control Biotechnology Co., LTD. Cells were cultured in DMEM/F12 medium supplemented with 10% (*v*/*v*) FBS and penicillin (100 U/mL) and streptomycin (100 µg/mL) at 37 °C with 5% CO_2_ [20].

### 2.5. Cell Experiments

Caco-2 cells were plated at 5 × 10^5^ cells/well in a twelve-well plate and cultured for 24 h at 37 °C with 5% CO_2_. Subsequently, different concentrations of SBE were added and incubated for 12 h. Then, 7500 µM H_2_O_2_ was added to model and treatment groups and incubated for 24 h at 37 °C with 5% CO_2_. Then, samples were collected for subsequent detection.

### 2.6. Cell Survival Rate

Caco-2 cells were plated at 5 × 10^5^ cells/well in a 96-well plate and cultured for 24 h at 37 °C with 5% CO_2_. Subsequently, different concentrations of SBE were added and incubated for 12 h. Then 7500 µM H_2_O_2_ was added to model and treatment groups and incubated for 24 h at 37 °C with 5% CO_2_. After that, 10 μL MTT solution (5 mg/mL) was added to each well and culture for a further 4 h. Then, the culture supernatant was carefully removed from the well, 100 μL DMSO was added to each well, and they were gently shaken on the shaker for 10 min. After, the crystals at the bottom of the well were fully dissolved to measure OD_490_.

### 2.7. Measurement of Oxidative Stress Biochemical Markers

Cells were collected according to the method in Section 2.5. ROS were detected using a DCFH-DA(2,7-dichlorofuorescin Diacetate) probe; an MDA kit, based on the reaction of MDA and thiobarbituric acid (TBA) to produce red products, was subsequently used. MDA in cell lysates was quantified by colorimetry. The SOD kit is based on superoxide, which can reduce WST-1 to produce soluble colored substances to detect superoxide, and the CAT kit detects cell catalase activity through color reaction. All detection steps were performed according to the kit instructions.

### 2.8. Quantitative Real-Time PCR

Cells were collected according to the method in Section 2.5. Then, the culture medium was discarded and washed with sterile PBS 1 or 2 times, and 1 mL Trizol was added to each well. The reaction was performed at room temperature for 5 min, and the mixture was mixed by blowing with a pipetting gun. After blowing, the suspension was collected into a 1.5 mL EP tube, then 200 µL chloroform was added, vortexed for 15 s, and left for 2 min. The samples were centrifuged at 12,000 *g* for 10 min at 4 °C. Carefully, the supernatant was transferred into another 1.5 mL EP tube, then isopropanol (500 µL) was added to each tube, mixed, and left at room temperature for 10 min. Then, the samples were centrifuged at 12,000 *g* for 10 min at 4 °C. The supernatant was removed, and 75% ethanol which had been precooled in advance was added to the EP tube. The EP tube was shaken by a vortex instrument for 3 to 5 s. Then, the samples were centrifuged at 7500 *g* for 5 min at 4 °C. The supernatant was discarded and allowed to dry at room temperature before dissolving in DEPC water to obtain RNA. A total of 1000 ng of RNA was added to 1 µL of Anchored Oligo (dT) 18 primer and RNase-free water for a total volume of 9 µL, and cDNA was obtained by reverse transcription. Quantitative real-time reactions were performed with 2 µL cDNA, 0.5 µL upstream and downstream primers, 10 µL Mix, and 7 µL ddH_2_O. The primer sequences of Rt-qPCR are shown in Table 1.

### 2.9. Western Blot

Cells were seeded at a density of 5 × 10^5^ cells/well in Coring cell culture dishes for 24 h before the treatment. After the corresponding treatment, cells were lysed with lysis buffer and then the cell suspension was intermittently shocked and the supernatants collected from centrifugation (15 min, 12,000 rpm, 4 °C). In brief, a BCA protein assay kit was employed to determine the protein concentrations. After mixing with 2 × SDS-PAGE sample buffer, an equal amount of protein (20 µg) was separated on a 10% (*w*/*v*) SDS-PAGE gel, and proteins were transferred onto PVDF membranes, which were blocked in a 5% skimmed milk powder buffer (room temperature, 2 h) before incubating the membranes with the corresponding primary antibodies (Nrf2 and Keap1) overnight at 4 °C. The samples were then rinsed with 1 × TBST three times, followed by incubation with a secondary antibody for 60 min at room temperature. To visualize the protein bands, the PVDF membrane was placed flat in the Image Quant LAS 500 imaging system and the ECL luminescent chromogenic solution was applied dropwise to the membrane surface. The grayscale values of the protein bands were measured with Image Pro plus 6.0, and the grayscale values of the protein bands to be measured were compared with the grayscale values of the internal reference protein to calculate the relative expression of the target protein. All protein bands were normalized to GAPDH [21,22].

### 2.10. Data Analysis

Three replicates of the experiment were used to generate each set of data, which were then analyzed using one-way ANOVA and mapping in Graphpad 8.0.2.

## 3. Results

### 3.1. Oxidative Stress Preventive Effect of SBE

Our previous study indicated that SBE has repaired oxidative stress effect, so to evaluate the preventive effect of SBE regarding oxidative stress damage, we pretreated Caco-2 cells with various concentrations of SBE and incubated for 12 h before subjecting them to 24 h of H_2_O_2_ exposure to determine the contents of ROS, MDA, SOD, and CAT.

**Figure 1:**
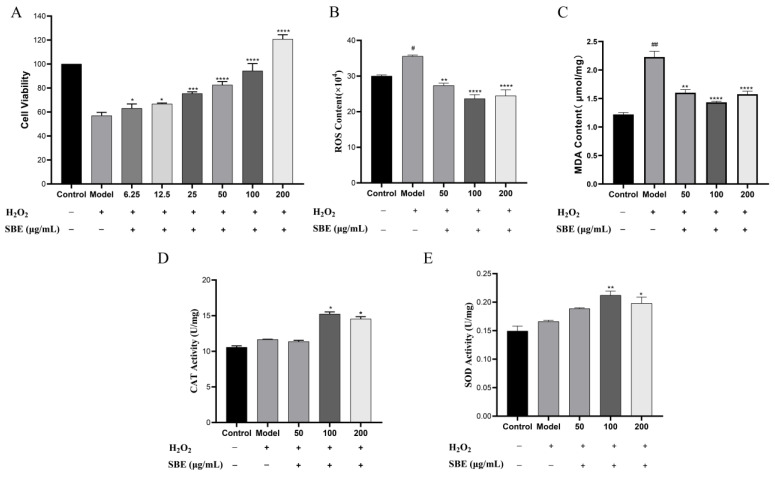
Effect of SBE on intracellular ROS production, MDA content, and SOD and CAT activity. (**A**) Cell viability; (**B**) ROS content; (**C**) MDA content; (**D**) CAT activity; (**E**) SOD activity. The values are expressed as the mean ± SD (n ≥ 3 per group). Significance between groups was analyzed using one-way ANOVA (compared with the control group, # *p* < 0.05, ## *p* < 0.01; compared with the model group, * *p* < 0.05, ** *p* < 0.01, *** *p* < 0.001, **** *p* < 0.0001).

The results demonstrated that the SBE treatment group could improve the cell survival rate in a dose-dependent relationship (Figure 1A). Then we selected SBE with concentrations of 50, 100, and 200 μg/mL to evaluate the effect preventing oxidative stress in cells. As shown in the Figure 1B,C, compared with the model group, we found that SBE treatment groups had scavenging effects on ROS and MDA, especially at 100 μg/mL.

Later, we measured the enzyme activity of CAT and SOD. The results revealed that the enzyme activity of CAT and SOD in the model group was increased compared with the control group, indicating that, when Caco-2 cells were stimulated by H_2_O_2_, the cells would produce CAT and SOD to fend off oxidative stress. Compared with model group, we found that the enzyme activity of CAT and SOD was augmented after SBE pre-protection treatment for 12 h, especially at 100 μg/mL (Figure 1D,E).

Finally, we found that SBE’s effect of preventing oxidative stress was able to decrease the content of ROS in Caco-2 cells, as well as reduce the production of lipid peroxide MDA and then increase the enzymatic activity of SOD and CAT. This was in response to our investigation into the preventive effect of SBE on oxidative damage in Caco-2 cells. These findings imply that SBE significantly enhances the prevention of oxidative stress.

### 3.2. Time–Effect Relationship of SBE Preventing Oxidative Stress

After we investigated the dose–effect relationship of SBE’s effect of preventing oxidative stress, we found that the SBE treatment group with 100 μg/mL had the best scavenging effect on ROS and MDA, as well as the highest enzyme activity of antioxidant enzymes. Therefore, the concentration of 100 μg/mL was selected to investigate the time–effect relationship of SBE’s prevention of oxidative stress.

**Figure 2:**
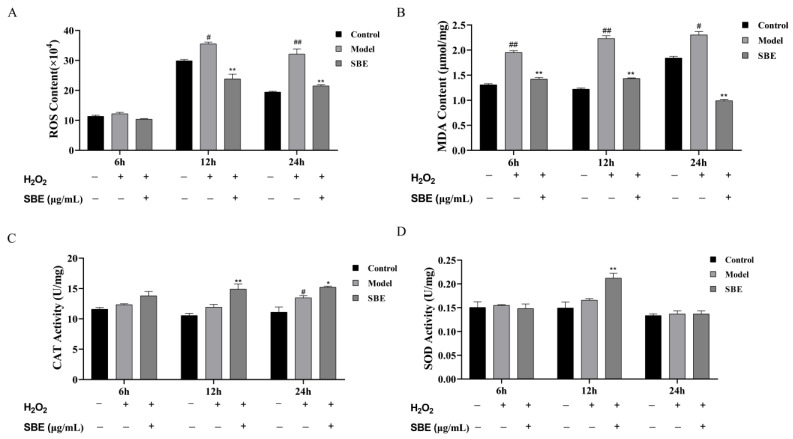
Time–effect relationship of preventing oxidative stress effect of SBE. (**A**) ROS content; (**B**) MDA content; (**C**) CAT activity; (**D**) SOD activity. The values are expressed as the mean ± SD (n ≥ 3 per group). Significance between groups was analyzed using one-way ANOVA (compared with the control group, ^#^ *p* < 0.05, ^##^ *p* < 0.01; compared with the model group, * *p* < 0.05, ** *p* < 0.01).

Compared with the model group, our results showed that pretreatment with SBE could reduce the content of ROS and MDA produced by oxidative stress in Caco-2 cells after 6 h, 12 h, and 24 h. In particular, the content of ROS can be significantly reduced at 12 h and 24 h (Figure 2A). SBE played an important role in the clearance of MDA within a very short pre-protection time, such as 6 h (Figure 2B), and in the time–effect relationship.

When we investigated the effect of SBE treatment time on the antioxidant enzyme activities, the results showed that either short or extended pre-protection time affected the activities of antioxidant enzymes. When Caco-2 cells were pre-protected with SBE for 6 h, there was no significant effect on the activities of CAT and SOD, while after pre-protecting with SBE for 12 h the enzyme activities of CAT and SOD were significantly increased (Figure 2C,D). However, compared with the model group, only the enzyme activity of CAT was significantly elevated in Caco-2 cells following a 24 h pre-protective treatment with SBE.

### 3.3. Effect of SBE on Nrf2 and Keap1 Expression

A crucial mechanism for oxidation and anti-oxidation in the body is Nrf2-Keap1-ARE signaling, and H_2_O_2_ damage will trigger a cascade of related genes in this system [23,24]. This pathway’s main regulator, Nrf2, can activate and promote the production of genes that are cytoprotective and antioxidative, which are essential regulators of intracellular redox homeostasis and oxidative stress network responses [25]. Nrf2 lessens the harm that reactive oxygen species and unsaturated electrons cause to cells by stabilizing cells, maintaining redox body reactions, and inducing and controlling the constitutively induced expression of a number of antioxidant proteins, which helps to maintain homeostasis [25,26,27]. Normally, Nrf2 and Keap1 bind in the cytoplasm, which is not activated. The binding between Nrf2 and Keap1 is unstable when cells experience oxidative stress. So, Nrf2 is released and transferred to the nucleus to bind with ARE, activating the transcription of downstream genes, and then translated to a series of related proteins to play a role in preventing oxidative stress.

Having demonstrated the antioxidant effect of SBE, we evaluated whether the antioxidant effect of SBE was mediated through the Nrf2-Keap1-ARE signaling pathway by examining the gene expression of *NFE2L2* and *KEAP1*. In accordance with the results of the earlier cell tests, we selected the concentrations (50, 100, and 200 μg/mL) of SBE along with the pre-protection time for 12 h. Consequently, the genes expression of *NFE2L2* and *KEAP1* and the protein expression of Nrf2 and Keap1 were detected.

**Figure 3:**
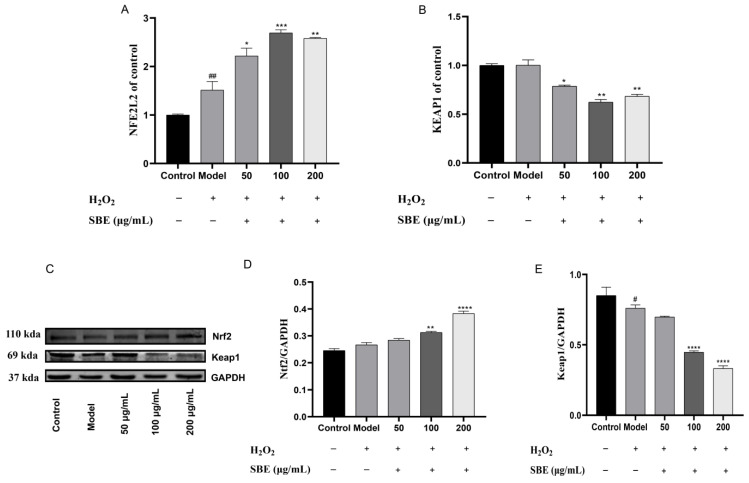
Effect of SBE on Nrf2 and Keap1 mRNA and protein expression levels in cells. GAPDH was used as an internal control. (**A**) Expression of NFE2L2 gene; (**B**) Expression of KEAP1 gene; (**C**) Western Blot analysis of Nrf2 and keap1; (**D**) Expression of Nrf2 protein; (**E**) Expression of Keap1 protein. The values are expressed as the mean ± SD (n ≥ 3 per group). Significance between groups was analyzed using one-way ANOVA (compared with the control group, ^#^ *p* < 0.05, ^##^ *p* < 0.01; compared with the model group, * *p* < 0.05, ** *p* < 0.01, *** *p* < 0.001, **** *p* < 0.0001).

Compared with the model group, our results showed that SBE pretreatment could considerably raise *NFE2L2* mRNA expression levels, with the 100 μg/mL concentration group showing the highest expression level compared to the other two groups (Figure 3A). The levels of *KEAP1* mRNA expression showed similar outcomes. *KEAP1* mRNA expression levels decreased in the SBE treatment group at various dosages; however, the reduction effect was more pronounced in the 100 μg/mL group (Figure 3B).

Successively, the protein expressions of Nrf2 and Keap1 were analyzed based on the Western blot in Caco-2 cells (Figure 3C). Comparing with the model group, we found that the SBE treatment groups significantly enhanced the protein expression level of Nrf2 while decreasing the protein expression level of Keap1. Meanwhile, the protein expression level of Nrf2 increased and that of Keap1 decreased in the 100 μg/mL group better than in the other SBE treatment groups, which is consistent with the trend of SBE regulating the gene expression of *NFE2L2* and *KEAP1* (Figure 3D,E). The results reveal that SBE greatly boosted the protein expression of Nrf2, a crucial antioxidant transcription factor, and has a regulatory influence on Keap1 protein expression.

### 3.4. Effect of SBE on the Expression of Genes Downstream of the Antioxidant Pathway

Having demonstrated that SBE regulates the key proteins in the Nrf2-Keap1-ARE signaling pathway, after that we examined the expression of genes downstream of this pathway: *HMOX1*, *NQO1*, *SOD1*, *CAT*, and *GPX1*.

Heme oxygenase (HO-1) is a phase II detoxifying enzyme that catalyzes the formation of biliverdin, carbon monoxide, and iron from heme. Biliverdin has the ability to transform into bilirubin, a potent free radical scavenger, making it an important cytoprotective factor downstream of the antioxidant pathway Nrf2-Keap-ARE [28,29,30,31]. Quinone hydroxyl oxidoreductase 1 (NQO1) is regulated by Nrf2 to reduce quinones to hydroquinone, thereby promoting their excretion. In the absence of NQO1, quinones form semihydroquinones through electron reduction reactions, and semihydroquinones generate ROS through redox cycling [32]. NQO1, hence, contributes significantly to a decrease in ROS.

**Figure 4:**
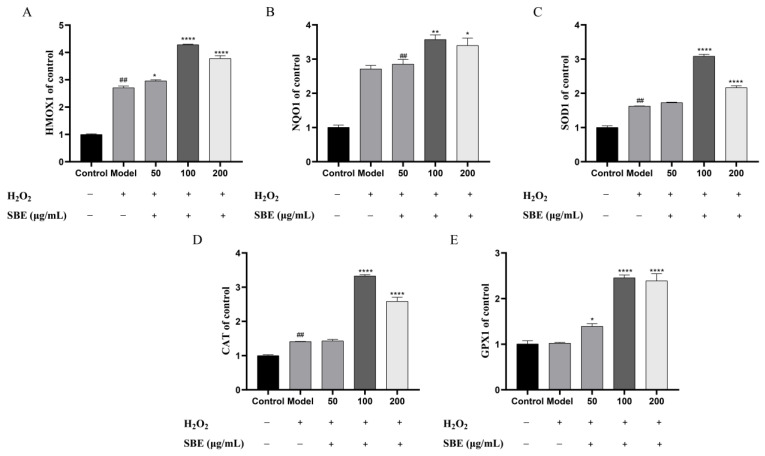
Effects of SBE on genes downstream of the antioxidant pathway. (**A**) Expression of HMOX1 gene; (**B**) Expression of NQO1 gene; (**C**) Expression of SOD1 gene; (**D**) Expression of CAT gene; (**E**) Expression of GPX1 gene; The values are expressed as the mean ± SD (n ≥ 3 per group). Significance between groups was analyzed using one-way ANOVA (compared with the control group, ^##^ *p* < 0.01; compared with the model group, * *p* < 0.05, ** *p* < 0.01, **** *p* < 0.0001).

Our results showed that the mRNA expression levels of HMOX1 and NQO1 were significantly higher compared with the model group, the 100 and 200 μg/mL groups having an especially significant effect (Figure 4A,B). These results indicated that pretreatment with SBE could protect cells from oxidative stress.

Furthermore, we evaluated whether SBE was able to enhance the oxidative stress preventive effect of Caco-2 cells by enhancing the mRNA expression level of the antioxidant enzymes. Among them, SOD is an important antioxidant enzyme in the body to remove oxygen free radicals, CAT can decompose H_2_O_2_ to produce H_2_O and O_2_, and GSH-Px can help maintain the redox balance of cells in the organism and can break down peroxides [33]. We found that SBE could increase the mRNA expression levels of *SOD1*, *CAT*, and GPX1, especially in the 100 and 200 μg/mL SBE treatment groups, where the effects were considerably greater than the model group (Figure 4C–E).

## 4. Discussion

The oxidation–antioxidant balancing system is made up of the rate at which reactive oxygen species are produced and the pace at which antioxidants are cleared in physiological conditions. This equilibrium will be break down when the body is driven by oxidation, resulting in the buildup of ROS and an imbalance between the oxidative and antioxidant systems [34]. Consequently, ROS detection can serve as an indirect indicator of the development of oxidative stress [35]. Lipid oxidation produces MDA, which has an impact on the function of essential enzymes in mitochondria [36]. The degree of lipid oxidation in the body may therefore be indirectly reflected by the level of MDA. The antioxidant enzyme system (SOD and CAT) serves as the body’s first line of defense against oxidative stress [37]. Therefore, in this work, the oxidative stress preventive capability of SBE was evaluated by detecting ROS, MDA, SOD, and CAT based on the H_2_O_2_-induced Caco-2 cells model.

According to our results, it was discovered that, after Caco-2 cells were treated for 12 h with SBE at three different doses (50, 100, and 200 μg/mL), the amounts of intracellular ROS and MDA were decreased, and in a dose-dependent relationship (Figure 1A,B). Simultaneously, pretreatment with SBE at a high dose (100 and 200 μg/mL) could boost SOD and CAT enzyme activity, and in a dose-dependent relationship (Figure 1C,D). In our previous work, we have proven that the main functional components of SBE playing a role in the repair of oxidative damage were polysaccharides, alkaloids, and polyphenols by analysis of the primary constituents of SBE [18]. Liao [38] showed that *Ischnoderma Resinosum* polysaccharides had a strong antioxidant activity to protect injured cells, and the protective effect on cells was dose-dependent.

Our results revealed that the effect of the high-concentration group was inferior to that of the low-concentration group under the conditions of short-term treatment, which may be related to the drug’s transmembrane transport, including its degree of dissociation, lipid solubility, and any potential distinction between its intracellular and extracellular distribution. Guan [39] found that protocatechuic acid enhanced the protective effect on nerve cells through the activities of intracellular antioxidant enzymes SOD and CAT. Based on the above analysis, we concluded that SBE has a preventive effect on oxidative stress related to its polysaccharide and polyphenol content.

Biosystems have developed an adaptive defense system to resist oxidative and omnipresent chemical stresses, consisting of dozens of cytoprotective genes, which can produce adaptations to different stressors. These genes are required as a cofactor for some of the various systems, which include glutathione- and thioredoxin-based antioxidant metabolic pathways, lipid metabolism, drug-resistance protein transporters, and so on. Most of these cytoprotective genes contain antioxidant response element (ARE) in the 5′ upstream regulatory region cis-acting sequences that regulate the transcription through binding to basic structural domains and leucine zipper (bZIP) transcription factors (TFs); the one that stands out is Nrf2 (NF-E2 p45-related factor 2). Conventional wisdom suggests that the Kelch-like ECH-associated protein 1 (Keap1) is a redox/electrophile-sensitive negative regulator of the Nrf2/ARE signaling pathway, which, in turn, mediates the expression of hundreds of genes involved in the cytoprotective systems [40,41,42].

**Figure 5:**
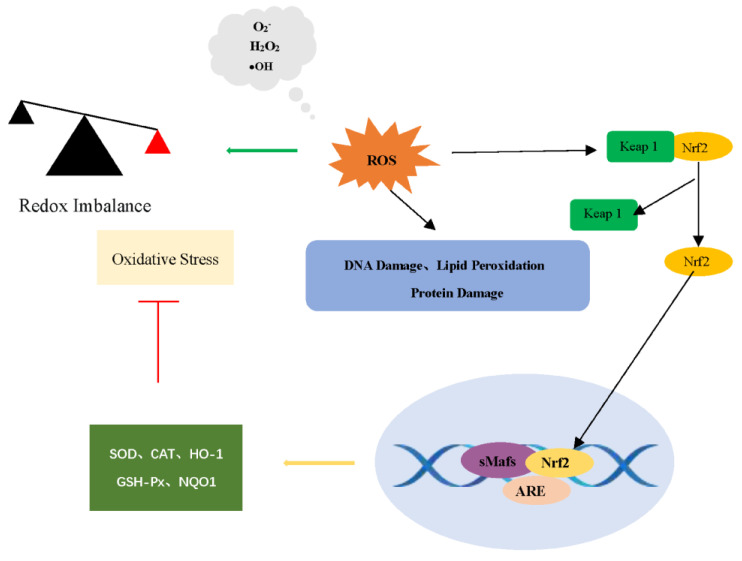
Nrf2-Keap1-ARE signaling pathway.

As the most dominant nuclear transcription factor, Nrf2 plays a major role in mediating cellular defense against oxidative stress and inflammation. According to related studies, the Keap1-Nrf2-ARE signaling pathway is an important defensive transduction pathway for the body against internal and external oxidative stress [23,24]. The transcription factor Nrf2 is involved in regulating the expression of downstream antioxidant genes and cytoprotective genes [43]. Under physiological conditions, Kelch-like ECH-associated protein 1 (Keap1) retains Nrf2 in the cytoplasm and promotes polyubiquitination of Nrf2 by recruiting the E3 ligase Cul3, thereby labeling Nrf2 for degradation via a proteasome-dependent mechanism [44]. At this time, only a very small fraction of free Nrf2 is transferred to the nucleus, where it binds to the GCTGAGTCA site on the antioxidant response element (ARE) sequence of cytoprotective genes and maintains the basal expression of cytoprotective genes. When stimulated, Keap1-mediated ubiquitination and protein degradation of Nrf2 is diminished, and the transfer of Nrf2 into the nucleus is increased, thereby up-regulating the expression of cytoprotective genes and increasing the resistance of cells to oxidative stress [45,46]. Activated Nrf2 translocates to the nucleus to bind to the ARE, which is essential for the transcriptional activation of antioxidant genes such as *NQO1* and *HMOX1*. In addition, this signaling pathway has a negative feedback mechanism, wherein the activated Nrf2 gradually restores the oxidation–reduction state of the cell to a certain level by up-regulating the level of cytoprotective proteins [41,44,47]. Therefore, the regulation of Nrf2 and Keap1 plays an important role in the antioxidant action (Figure 5).

In our investigations, we found that, compared to the model group, the mRNA expression level of *NFE2L2* was significantly up-regulated while the mRNA expression level of *KEAP1* was significantly down-regulated with SBE treatment (Figure 3A,B). The experimental results of Western blotting further accurately reflects the protein expression levels, so we examined Nrf2 and Keap1 proteins, and the protein expression and gene expression trends were consistent (Figure 3D,E), which means the Nrf2-Keap1-ARE signaling pathway is activated in response to oxidative stress. Studies have shown that activation of Nrf2 and inhibition of Keap1 can exert antioxidant effects [48,49,50]. Further analysis of the downstream genes (*HMOX1*, *NQO1*, *SOD1*, *CAT*, *GPX1*) showed that downstream genes’ mRNA expression levels were up-regulated compared to the model group (Figure 4). Mittal et al. [51] found out by investigating the effect of andrographolide on H_2_O_2_-induced oxidative damage in HepG2 cells that andrographolide could mitigate oxidative damage by up-regulating Nrf2 and thus activating the expression of downstream antioxidant genes. Therefore, we speculate that SBE may be an activator of Nrf2. Other studies have confirmed that up-regulating the protein expression of the Nrf2 could activate downstream genes (*SOD*, *HMOX1*, *NQO1*, *GPX1*) to exert antioxidant effects [52,53,54]. Our results also showed that SBE could significantly up-regulate the protein expression of Nrf2 and decrease the protein expression of Keap1, which activates the Nrf2-Keap1-ARE pathway to exert effects preventing oxidative stress.

## 5. Conclusions

We concluded in this study that SBE exerted an effect preventing oxidative stress by considerably reducing the content of ROS and MDA and increasing the enzyme activity of SOD and CAT. SBE might boost the expression of downstream antioxidant genes in this system according to the identification of relevant genes and proteins in the Nrf2-Keap1-ARE pathway, suggesting that SBE exerts an effect preventing oxidative stress through this pathway.

## Figures and Tables

**Table 1:** Sequence of RT-qPCR primers.

Gene		Primer Sequence (5′–3′)
*KEAP1*	F	CCTTCAGCTACACCCTGGAG
	R	AACATGGCCTTGAAGACAGG
*NFE2L2*	F	AGACAAACATTCAAGCCGCT
	R	CCATCTCTTGTTTGCTGCAG
*HMOX1*	F	CAGTCTTCGCCCCTGTCTAC
	R	GCTGGTGTGTAGGGGATGAC
*NQO1*	F	AGTGCAGTGGTGTGATCTCG
	R	GGTGGAGTCACGCCTGTAAT
*SOD1*	F	GAAGGTGTGGGGAAGCATTA
	R	GAAGGTGTGGGGAAGCATTA
*CAT*	F	ACATGGTCTGGGACTTCTGG
	R	CTTGGGTCGAAGGCTATCTG
*GPX1*	F	AACCAGTTTGGGCATCAGG
	R	GTTCACCTCGCACTTCTCG
*GAPDH*	F	GACCCCTTCATTGACCTCAAC
	R	CATACCAGGAAATGAGCTTG

## Data Availability

Data is contained within the article.

## References

[1] Roberts C.K., Sindhu K.K. (2009). Oxidative stress and metabolic syndrome. Life Sci..

[2] Diaz de Barboza G., Guizzardi S., Moine L., Tolosa de Talamoni N. (2017). Oxidative stress, antioxidants and intestinal calcium absorption. World J. Gastroenterol..

[3] Ushio-Fukai M., Ash D., Nagarkoti S., Belin de Chantemele E.J., Fulton D.J.R., Fukai T. (2021). Interplay Between Reactive Oxygen/Reactive Nitrogen Species and Metabolism in Vascular Biology and Disease. Antioxid. Redox Signal..

[4] Jayawardena T.U., Wang L., Sanjeewa K.K.A., Kang S.I., Lee J.S., Jeon Y.J. (2020). Antioxidant Potential of Sulfated Polysaccharides from Padina boryana; Protective Effect against Oxidative Stress in In Vitro and In Vivo Zebrafish Model. Mar. Drugs.

[5] Prasad S., Gupta S.C., Tyagi A.K. (2017). Reactive oxygen species (ROS) and cancer: Role of antioxidative nutraceuticals. Cancer Lett..

[6] Zahari A., Ablat A., Sivasothy Y., Mohamad J., Choudhary M.I., Awang K. (2016). In vitro antiplasmodial and antioxidant activities of bisbenzylisoquinoline alkaloids from Alseodaphne corneri Kosterm. Asian Pac. J. Trop. Med..

[7] Ktari N., Bkhairia I., Nasri R., Ben Abdallah Kolsi R., Ben Slama-Ben Salem R., Ben Amara I., Zeghal N., Ben Salah B., Ben Salah R., Nasri M. (2017). Zebra blenny protein hydrolysates as a source of bioactive peptides with prevention effect against oxidative dysfunctions and DNA damage in heart tissues of rats fed a cholesterol-rich diet. Food Res. Int..

[8] Fiedor J., Burda K. (2014). Potential Role of Carotenoids as Antioxidants in Human Health and Disease. Nutrients.

[9] Lu C.C., Wei R.X., Deng D.H., Luo Z.Y., Abdulai M., Liu H.H., Kang B., Hu S.Q., Li L., Xu H.Y. (2021). Effect of different types of sugar on gut physiology and microbiota in overfed goose. Poult. Sci..

[10] Zhou X., Wang W., Wang C., Zheng C., Xu X., Ni X., Hu S., Cai B., Sun L., Shi K. (2019). DPP4 Inhibitor Attenuates Severe Acute Pancreatitis-Associated Intestinal Inflammation via Nrf2 Signaling. Oxid. Med. Cell. Longev.

[11] Ma Y., Xiong Y.L., Zhai J., Zhu H., Dziubla T. (2010). Fractionation and evaluation of radical-scavenging peptides from in vitro digests of buckwheat protein. Food Chem..

[12] Bhattacharyya A., Chattopadhyay R., Mitra S., Crowe S.E. (2014). Oxidative stress: An essential factor in the pathogenesis of gastrointestinal mucosal diseases. Physiol. Rev..

[13] Pérez S., Taléns-Visconti R., Rius-Pérez S., Finamor I., Sastre J. (2017). Redox signaling in the gastrointestinal tract. Free Radic. Biol. Med..

[14] Kong Y., Olejar K.J., On S.L.W., Chelikani V. (2020). The Potential of *Lactobacillus* spp. for Modulating Oxidative Stress in the Gastrointestinal Tract. Antioxidants.

[15] Wang C., Liu J., Su Y., Li M., Xie X., Su J. (2021). Complete Chloroplast Genome Sequence of Sonchus brachyotus Helps to Elucidate Evolutionary Relationships with Related Species of Asteraceae. Biomed. Res. Int..

[16] Xia D.Z., Yu X.F., Zhu Z.Y., Zou Z.D. (2011). Antioxidant and antibacterial activity of six edible wild plants (*Sonchus* spp.) in China. Nat. Prod. Res..

[17] Pan F.F., Zhang H.Y., Li X.M., Yang P.L., Zhang T.C., Luo X.G., Ma W.J. (2018). Effect of quality control on the total antioxidant capacity of the extract from Sonchus brachyotus DC. Int. J. Food Prop..

[18] Yang J., Zhou W.W., Shi D.D., Pan F.F., Sun W.W., Yang P.L., Li X.M. (2023). The Interaction between Oxidative Stress Biomarkers and Gut Microbiota in the Antioxidant Effects of Extracts from Sonchus brachyotus DC. in Oxazolone-Induced Intestinal Oxidative Stress in Adult Zebrafish. Antioxidants.

[19] Pan F.F., Li X.M., Zhang H.Y., Ma W.J., Yang P.L. (2018). Optimization of Extracting Process of Total Alkaloids from Sonchus brachyotus DC. by Response Surface Method. Sci. Technol. Food Ind..

[20] Martinez M.A., Ares I., Martinez M., Lopez-Torres B., Maximiliano J.E., Rodriguez J.L., Martinez-Larranaga M.R., Anadon A., Peteiro C., Rubino S. (2021). Brown marine algae Gongolaria baccata extract protects Caco-2 cells from oxidative stress induced by tert-butyl hydroperoxide. Food Chem. Toxicol..

[21] Hernandez-Valencia J., Garcia-Villa E., Arenas-Hernandez A., Garcia-Mena J., Diaz-Chavez J., Gariglio P. (2018). Induction of p53 Phosphorylation at Serine 20 by Resveratrol Is Required to Activate p53 Target Genes, Restoring Apoptosis in MCF-7 Cells Resistant to Cisplatin. Nutrients.

[22] Katsube R., Noma K., Ohara T., Nishiwaki N., Kobayashi T., Komoto S., Sato H., Kashima H., Kato T., Kikuchi S. (2021). Fibroblast activation protein targeted near infrared photoimmunotherapy (NIR PIT) overcomes therapeutic resistance in human esophageal cancer. Sci. Rep..

[23] Naguib S., Backstrom J.R., Gil M., Calkins D.J., Rex T.S. (2021). Retinal oxidative stress activates the NRF2/ARE pathway: An early endogenous protective response to ocular hypertension. Redox Biol..

[24] Jia R., Gu Z., He Q., Du J., Cao L., Jeney G., Xu P., Yin G. (2019). Anti-oxidative, anti-inflammatory and hepatoprotective effects of Radix Bupleuri extract against oxidative damage in tilapia (*Oreochromis niloticus*) via Nrf2 and TLRs signaling pathway. Fish Shellfish Immunol..

[25] Zheng Y.H., Yang J.J., Tang P.J., Zhu Y., Chen Z., She C., Chen G., Cao P., Xu X.Y. (2021). A novel Keap1 inhibitor iKeap1 activates Nrf2 signaling and ameliorates hydrogen peroxide-induced oxidative injury and apoptosis in osteoblasts. Cell Death Dis..

[26] Liang L., Luo M., Fu Y., Zu Y., Wang W., Gu C., Zhao C., Li C., Efferth T. (2013). Cajaninstilbene acid (CSA) exerts cytoprotective effects against oxidative stress through the Nrf2-dependent antioxidant pathway. Toxicol. Lett..

[27] Shokeir A.A., Hussein A.M., Barakat N., Abdelaziz A., Elgarba M., Awadalla A. (2014). Activation of nuclear factor erythroid 2-related factor 2 (Nrf2) and Nrf-2-dependent genes by ischaemic pre-conditioning and post-conditioning: New adaptive endogenous protective responses against renal ischaemia/reperfusion injury. Acta Physiol..

[28] Fernández-Mendívil C., Luengo E., Trigo-Alonso P., García-Magro N., Negredo P., López M.G. (2021). Protective role of microglial HO-1 blockade in aging: Implication of iron metabolism. Redox Biol..

[29] Kim W., Kim S.H., Jang J.H., Kim C., Kim K., Suh Y.G., Joe Y., Chung H.T., Cha Y.N., Surh Y.J. (2018). Role of heme oxygenase-1 in potentiation of phagocytic activity of macrophages by taurine chloramine: Implications for the resolution of zymosan A-induced murine peritonitis. Cell Immunol..

[30] Rivera-Pérez J., Martínez-Rosas M., Conde-Castañón C.A., Toscano-Garibay J.D., Ruiz-Pérez N.J., Flores P.L., Mera Jiménez E., Flores-Estrada J. (2020). Epigallocatechin 3-Gallate Has a Neuroprotective Effect in Retinas of Rabbits with Ischemia/Reperfusion through the Activation of Nrf2/HO-1. Int. J. Mol. Sci..

[31] Wang P., Zhao Y., Li Y., Wu J., Yu S., Zhu J., Li L., Zhao Y. (2019). Sestrin2 overexpression attenuates focal cerebral ischemic injury in rat by increasing Nrf2/HO-1 pathway-mediated angiogenesis. Neuroscience.

[32] Su X.L., Wang J.W., Jiang L., Huang X.M. (2021). In Synergistic Lethality between Beta-lapachone and Proliferating Cell Nuclear Antigen (PCNA) Inhibitor in NQO1-positive Cancer Cells. FASEB Journal..

[33] Su L., Zhang J., Gomez H., Kellum J.A., Peng Z. (2023). Mitochondria ROS and mitophagy in acute kidney injury. Autophagy.

[34] Schieber M., Chandel N.S. (2014). ROS Function in Redox Signaling and Oxidative Stress. Curr. Biol..

[35] Chouchani E.T., Pell V.R., Gaude E., Aksentijevic D., Sundier S.Y., Robb E.L., Logan A., Nadtochiy S.M., Ord E.N.J., Smith A.C. (2014). Ischaemic accumulation of succinate controls reperfusion injury through mitochondrial ROS. Nature.

[36] Dillard C.J., Litov R.E., Savin W.M., Dumelin E.E., Tappel A.L. (1978). Effects of exercise, vitamin E, and ozone on pulmonary function and lipid peroxidation. J. Appl. Physiol. Respir. Environ. Exerc. Physiol..

[37] Dandapat J., Chainy G.B., Rao K.J. (2003). Lipid peroxidation and antioxidant defence status during larval development and metamorphosis of giant prawn, Macrobrachium rosenbergii. Comp. Biochem. Physiol. C Toxicol. Pharmacol..

[38] Liao C., Wu L., Zhong W., Zheng Q., Tan W., Feng K., Feng X., Meng F. (2022). Cellular Antioxidant Properties of Ischnoderma Resinosum Polysaccharide. Molecules.

[39] Guan S., Zhang X.L., Ge D., Liu T.Q., Ma X.H., Cui Z.F. (2011). Protocatechuic acid promotes the neuronal differentiation and facilitates survival of phenotypes differentiated from cultured neural stem and progenitor cells. Eur. J. Pharmacol..

[40] Hayes J.D., Dinkova-Kostova A.T. (2014). The Nrf2 regulatory network provides an interface between redox and intermediary metabolism. Trends Biochem. Sci..

[41] Suzuki T., Yamamoto M. (2015). Molecular basis of the Keap1-Nrf2 system. Free Radic. Biol. Med..

[42] Ulasov A.V., Rosenkranz A.A., Georgiev G.P., Sobolev A.S. (2022). Nrf2/Keap1/ARE signaling: Towards specific regulation. Life Sci..

[43] Sardaro N., Della Vella F., Incalza M.A., Di Stasio D., Lucchese A., Contaldo M., Laudadio C., Petruzzi M. (2019). Oxidative Stress and Oral Mucosal Diseases: An Overview. In Vivo.

[44] Zhang W., Cheng C., Sha Z., Chen C., Yu C., Lv N., Ji P., Wu X., Ma T., Cheng H. (2021). Rosmarinic acid prevents refractory bacterial pneumonia through regulating Keap1/Nrf2-mediated autophagic pathway and mitochondrial oxidative stress. Free Radic. Biol. Med..

[45] Sajadimajd S., Khazaei M. (2018). Oxidative Stress and Cancer: The Role of Nrf2. Curr. Cancer Drug Targets.

[46] Gao B., Doan A., Hybertson B.M. (2014). The clinical potential of influencing Nrf2 signaling in degenerative and immunological disorders. Clin. Pharmacol..

[47] Ucar B.I., Ucar G., Saha S., Buttari B., Profumo E., Saso L. (2021). Pharmacological Protection against Ischemia-Reperfusion Injury by Regulating the Nrf2-Keap1-ARE Signaling Pathway. Antioxidants.

[48] Yin J., Ren W., Liu G., Duan J.L., Yang G., Wu L., Li T., Yin Y. (2013). Birth oxidative stress and the development of an antioxidant system in newborn piglets. Free. Radic. Res..

[49] Xu J., Zhou L., Weng Q., Xiao L., Li Q. (2019). Curcumin analogues attenuate Aβ25-35-induced oxidative stress in PC12 cells via Keap1/Nrf2/HO-1 signaling pathways. Chem.-Biol. Interact..

[50] Fakhri S., Pesce M., Patruno A., Moradi S.Z., Iranpanah A., Farzaei M.H., Sobarzo-Sinchez E. (2020). Attenuation of Nrf2/Keap1/ARE in Alzheimer’s Disease by Plant Secondary Metabolites: A Mechanistic Review. Molecules.

[51] Mittal S.P.K., Khole S., Jagadish N., Ghosh D., Gadgil V., Sinkar V., Ghaskadbi S.S. (2016). Andrographolide protects liver cells from H_2_O_2_ induced cell death by upregulation of Nrf-2/HO-1 mediated via adenosine A2a receptor signaling. Biochim. Biophys. Acta Gen. Subj..

[52] Wang C.M., Li Y.J., Li J.J., Zang Y.L., Cui X.H., Song M., Yang Q., Chen Y., Li Q., Cai W.Y. (2021). Shenlian extract attenuates TNF-α-induced ECV304 injury by regulating Nrf2 /Keap1 signaling pathway. China J. Chin. Mater. Medica.

[53] Dang R.Z., Wang M.Y., Li X., Wang H.Y., Li L., Wu Q.Y., Zhao J.T., Ji P., Zhong L.M., Julio L. (2022). Edaravone ameliorates depressive and anxiety-like behaviors via Sirt1/Nrf2/HO-1/ Gpx4 pathway. J. Neuroinflammation.

[54] Yu C.W., Chen H., Du D.H., Lv W.T., Li S.J., Li D.F., Xu Z.X., Gao M., Hu H.L., Liu D.C. (2021). β-Glucan from Saccharomyces cerevisiae alleviates oxidative stress in LPS-stimulated RAW264.7 cells via Dectin-1/Nrf2/HO-1 signaling pathway. Cell Stress Chaperones.

